# Bibliometric analysis of acupuncture for headache from 1974 to 2022: A scoping literature review based on international database

**DOI:** 10.1097/MD.0000000000034590

**Published:** 2023-08-04

**Authors:** Jin-Huan Yue, Ang Li, Xuan Cui, Xu-Chen Sun, Xiao-Ling Li, Xu Yang, Xiao Liu, Dan-Na Cao, Wei-Wei Zhao, Guan-Hu Yang, Brenda Golianu, Yang Wang, Sheng-Wang Han, Qin-Hong Zhang

**Affiliations:** a Shenzhen Frontier in Chinese Medicine Research Co., Ltd., Shenzhen, China; b Department of Acupuncture and Moxibustion, Shenzhen Jiuwei Chinese Medicine Clinic, Shenzhen, China; c Pharmaceutical Research and Development Co. Ltd., Beijing, China; d Graduate School of Heilongjiang University of Chinese Medicine, Harbin, China; e Division of CT and MRI, First Affiliated Hospital of Heilongjiang University of Chinese Medicine, Harbin, China; f Department of Pediatrics, First Affiliated Hospital of Heilongjiang University of Chinese Medicine, Harbin, China; g MSD R and D (China) Co., Ltd, Beijing, China; h Department of Specialty Medicine, Ohio University, Athens, OH; i Department of Anesthesia, Stanford University, CA; j Third Ward of Rehabilitation Department, Second Affiliated Hospital of Heilongjiang University of Chinese Medicine, Harbin, China.

**Keywords:** acupuncture, bibliometric analysis, CiteSpace, headache, hotspots, trends, VOSviewer

## Abstract

This study aimed to investigate the research hotspots and global trends of acupuncture in the treatment of headaches from 1974 to 2022. The Web of Science core collection database and literature related to acupuncture for headache treatment were retrieved. The CiteSpace (version 5.1.R8) and VOSviewer (version 1.6.19) software perform collaborative network analysis on the information of countries, academic institutions, authors, and co-occurrence network analysis on keywords, co-cited journals, and references. A total of 841 studies were included. Overall, the number of publications has increased over the past 5 decades. We identified and analyzed the countries, institutions, authors, and journals that were most active in the domain of acupuncture treatment for headaches. The most productive countries were the United States and China. Chengdu University of Traditional Chinese Medicine was the most productive institution and Linde Klaus was the most productive author. Cephalalgia was the most productive and co-cited journal, whereas Lancet had the highest impact factor. The research hotspots mainly focus on headache, migraine, tension headache, electroacupuncture, and acupuncture. Research trends have mainly focused on acupuncture therapy and its curative effects, migraine without aura, paroxysmal migraine, and the mechanism of acupuncture treatment. The main research hotspots and frontier trends were the therapeutic effect and mechanism of acupuncture for headaches. The mechanism of acupuncture in the treatment of headache mainly focused on the neural mechanism by multimodal MRI.

## 1. Introduction

Headache is one of the most common neurological disorders around the world.^[1]^ Such disorders often result in heavy healthcare burdens and economic costs owning to their high incidence and disability.^[1–3]^ According to the 2019 Global Burden of Disease Study, headache is the third leading cause of disability globally, resulting in an annual disability of 46.6 million people worldwide, accounting for 5.4% of all disabilities.^[4]^

Acupuncture is a traditional Chinese medicine treatment modality. It has been used to treat a variety of pain disorders, such as chronic pain,^[5,6]^ and postoperative pain.^[7]^ Various studies on acupuncture for headache have also been conducted, including the influence of acupuncture on the frequency of headache in patients with headache disorders,^[8]^ the effect of acupuncture for migraine,^[9,10]^ and acupuncture on tension-type headache,^[11]^ which have provided evidence for the benefit of acupuncture in the treatment of headache. Although considerable progress has been made, there is still much room for exploration, and a comprehensive bibliometric analysis is required. In this study, a comprehensive summary of research on acupuncture treatment for headaches was made, revealing research hotspots and frontier trends in this field.

Bibliometrics refers to the cross-science of quantitative analysis of knowledge resources and their associations using mathematical, statistical, and other metrological research methods that are widely used in the study of a large amount of scientific research data.^[12,13]^ This analytical approach explores the research hotspots and frontier trends in the field of acupuncture treatment for headaches using CiteSpace (version 5.1.R8) and VOSviewer software (version 1.6.19). By interpreting the structure and information in the network atlas, scholars can quickly and accurately grasp research hotspots and trends in the related fields.^[14]^

In this study, we performed a bibliometric analysis of published studies related to acupuncture treatment for headaches in the Web of Science Core Collection (WOSCC) since 1974. To the best of our knowledge, no bibliometric study has been conducted on this topic. This study provides insights into the literature on acupuncture therapy for headaches in accordance with the co-occurrence network of countries, academic institutions, authors, journals, and keyword clustering from 1974 to 2022.

## 2. Methods

### 2.1. Study design

In this study, we retrospectively performed bibliometric analyses based on WOSCC database. Microsoft Excel 2022, CiteSpace 5.1.R8 (School of Computer and Information Science, Drexel University, United States) and VOSviewer 1.6.19 (Centre for Scientific and Technological Research, Leiden University, Netherlands) softwares were used to analyze the number of publications, countries, institutions, authors, keywords, and references, as well as their associations.

### 2.2. Data acquisition

In this study, all literature was obtained from the WOSCC database on November 26, 2022. The literature types were “Article” and “Review” in English. The search terms used for retrieval in this study were “TS=[(‘Acupuncture’ OR ‘manual acupuncture’ OR ‘scalp acupuncture’ OR ‘electroacupuncture’ OR ‘auricular acupuncture’) AND (‘headache’)]”. We exported the “full record and all references” in plain text format. In total, 841 articles were published between 1974 and 2022.

### 2.3. Eligibility criteria

In this study, the inclusion criteria were associated literature on acupuncture for headaches. The exclusion criteria were editorial materials, proceedings papers, corrections, meeting abstracts, and new items.

### 2.4. Literature selection

Two authors independently selected the literature by scanning the titles and abstracts. All literature selections were performed according to the eligibility criteria. Any divergence between the 2 authors was resolved by a third author through discussion, and consensus was reached.

### 2.5. Data analysis

In this study, CiteSpace 5.1.R8 and VOSviewer 1.6.19 softwares were used to perform the bibliometric analysis, and Microsoft Excel was used to draw the trend chart of annual publications from 1974 to 2022. We used VOSviewer to conduct collaborative network analyses of countries, academic institutions, authors, journals, and keyword co-occurrence. In addition, we utilized CiteSpace to conduct keyword clustering and citation burst analyses.

The parameters of the CiteSpace software were as follows: Time slicing (1974–2022), the number of years per slice was 5; Each node type was selected at a time with node type (country, institution, author, keyword, cited reference, and cited journal); Node threshold (topn = 50), and; Pruning (pathfinder) with pruning sliced networks. The detailed information is available at http://cluster.cis.drexel.edu/~cchen/citespace/ and https://www.vosviewer.com/.

## 3. Results

### 3.1. Literature selection

**Figure 1.**
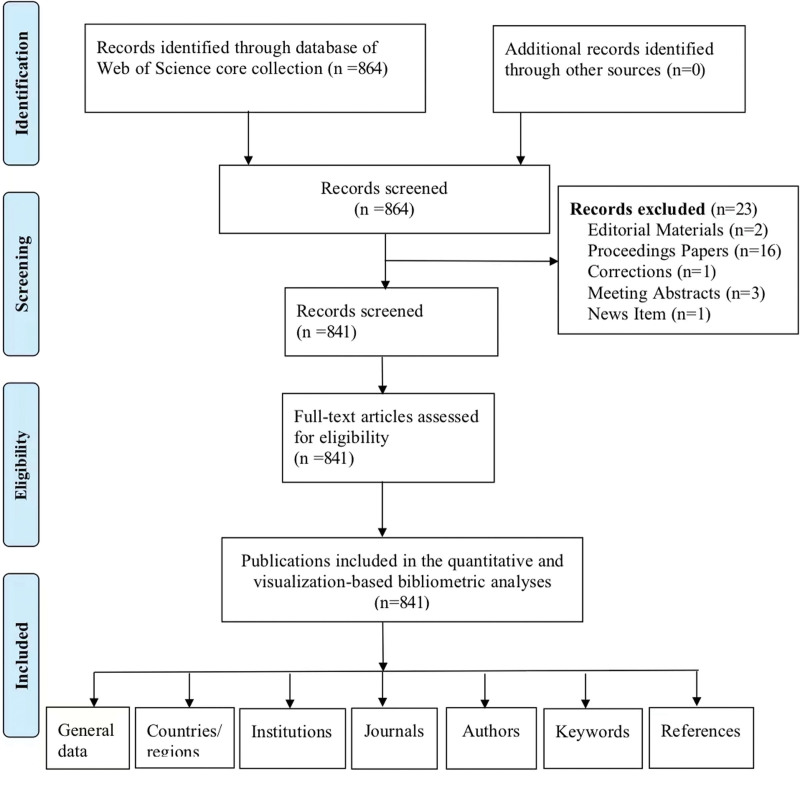
Procedure of study selection.

A total of 864 records were identified in the WOSCC database (Fig. 1). Of these, 23 records were excluded because of editorial materials (n = 2), proceedings papers (n = 16), corrections (n = 1), meeting abstracts (n = 3), or new items (n = 1). Finally, 841 eligible studies were included in the quantitative and visualization-based bibliometric analyses (Fig. 1).

### 3.2. Analysis of annual publications

**Figure 2.**
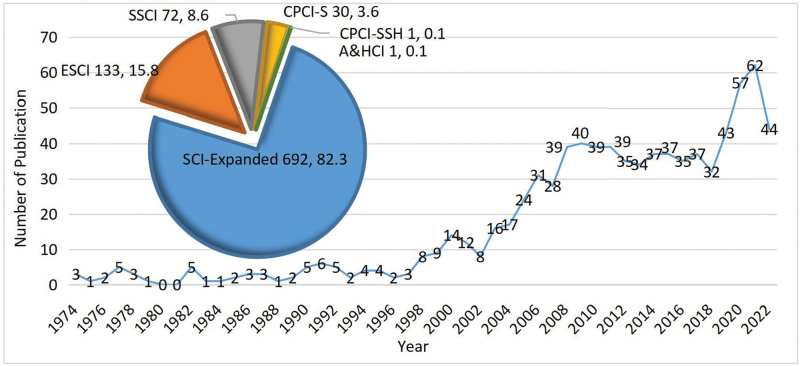
The number and trend of annual publications.

Annual publications on acupuncture treatment for headaches are presented in Figure 2. Overall, as can be seen from the figure, there was an increasing trend in publications. The number of publications fluctuated over the years, with a fitting curve index of y = 13.914 ln(x) to 23.887. The first such study was published in 1974. From 1974 to 1997, the number of annual publications increased to 6, with an average number of publications of 2.67 each year. From 1998 to 2008, the number of published articles increased with an average annual number of 18.73 publications. From 2009 to 2017, this figure fluctuated, with 37.00 publications per year. From 2018 to 2022, the number of publications has increased rapidly, peaking in 2021. Articles published in 2022 were incomplete because the search date was November 26, 2022. However, the annual literature trend and the fitting curve formula can predict future publication growth trends in this field.

### 3.3. Analysis of countries

**Figure 3.**
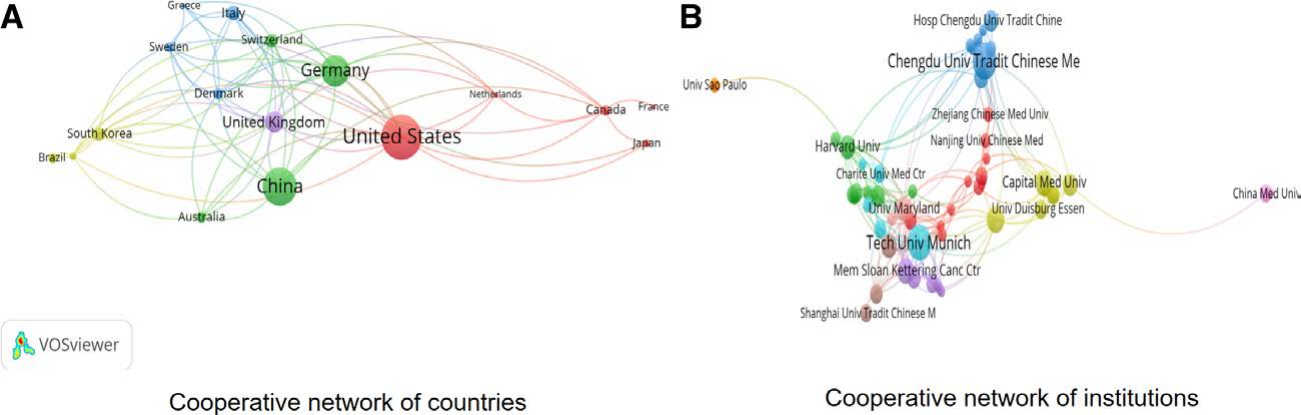
Cooperative network of authors (A) and institutions (B).

VOSviewer constructed a cooperative network of countries with 17 nodes and 69 links to acupuncture for headaches, as shown in Figure 3A. Each node represents 1 country. The size of each node indicated the number of publications. Links between nodes indicate cooperation, with wider links indicating closer collaboration.

The top 10 most productive countries are listed in Table 1. The most productive country was the United States (235 articles, 27.94%), followed by China (178 articles, 21.17%) and Germany (133 articles, 15.81%). The remaining countries included United Kingdom (71 articles), Italy (41 articles), Switzerland (35 articles), South Korea (34 articles), Denmark (28 articles), Australia (27 articles), and Canada (25 articles). China had the highest centrality (0.51), followed by Germany (0.43), the United States (0.19), and United Kingdom (0.10). This finding implies that China and the United States play essential roles in the research domain of acupuncture for headaches through their own international collaborative networks.

**Table 1:** Top ten productive countries and institutions.

Ranking	Country	Frequency (articles)	Centrality	Ranking	Institution	Frequency (articles)	Centrality
1	United States	235	0.19	1	Chengdu Univ Tradit Chinese Med	44	0.17
2	China	178	0.51	2	Tech Univ Munich	38	0.16
3	Germany	133	0.43	3	Beijing Univ Chinese Med	23	0.11
4	United Kingdom	71	0.10	4	Mem Sloan Kettering Canc Ctr	20	0.05
5	Italy	41	0.06	5	Capital Med Univ	19	0.09
6	Switzerland	35	0.02	6	Univ Maryland	18	0.02
7	South Korea	34	0.07	7	Harvard Univ	16	0.09
8	Denmark	28	0.01	8	China Acad Chinese Med Sci	15	0.06
9	Australia	27	0.06	9	Univ York	15	0.01
10	Canada	25	0.01	10	Sichuan Univ	13	0.05

### 3.4. Analysis of institutions

VOSviewer built a cooperative network of institutions with 64 nodes and 109 links to acupuncture for headaches, as shown in Figure 3B. We analyzed only institutions with at least 5 eligible publications. Each node represents an institution and its size indicates the number of published studies. The links between nodes suggest collaboration, with a wider link indicating closer collaboration.

The top 10 productive institutions are listed in Table 1. They were Chengdu University of Traditional Chinese Medicine (44 articles), Technical University of Munich (38 articles), Beijing University of Chinese Medicine (23 articles), Memory Sloan Kettering Cancer Center (20 articles), Capital Medical University (19 articles), University of Maryland (18 articles), Harvard University (16 articles), China Academy of Chinese Medical Sciences (15 articles), the University of York (15 articles), and Sichuan University (13 articles). Chengdu University of Traditional Chinese Medicine had the highest centrality (0.17), followed by the Technical University of Munich (0.16), and Beijing University of Chinese Medicine (0.11).

### 3.5. Analysis of authors

**Figure 4.**
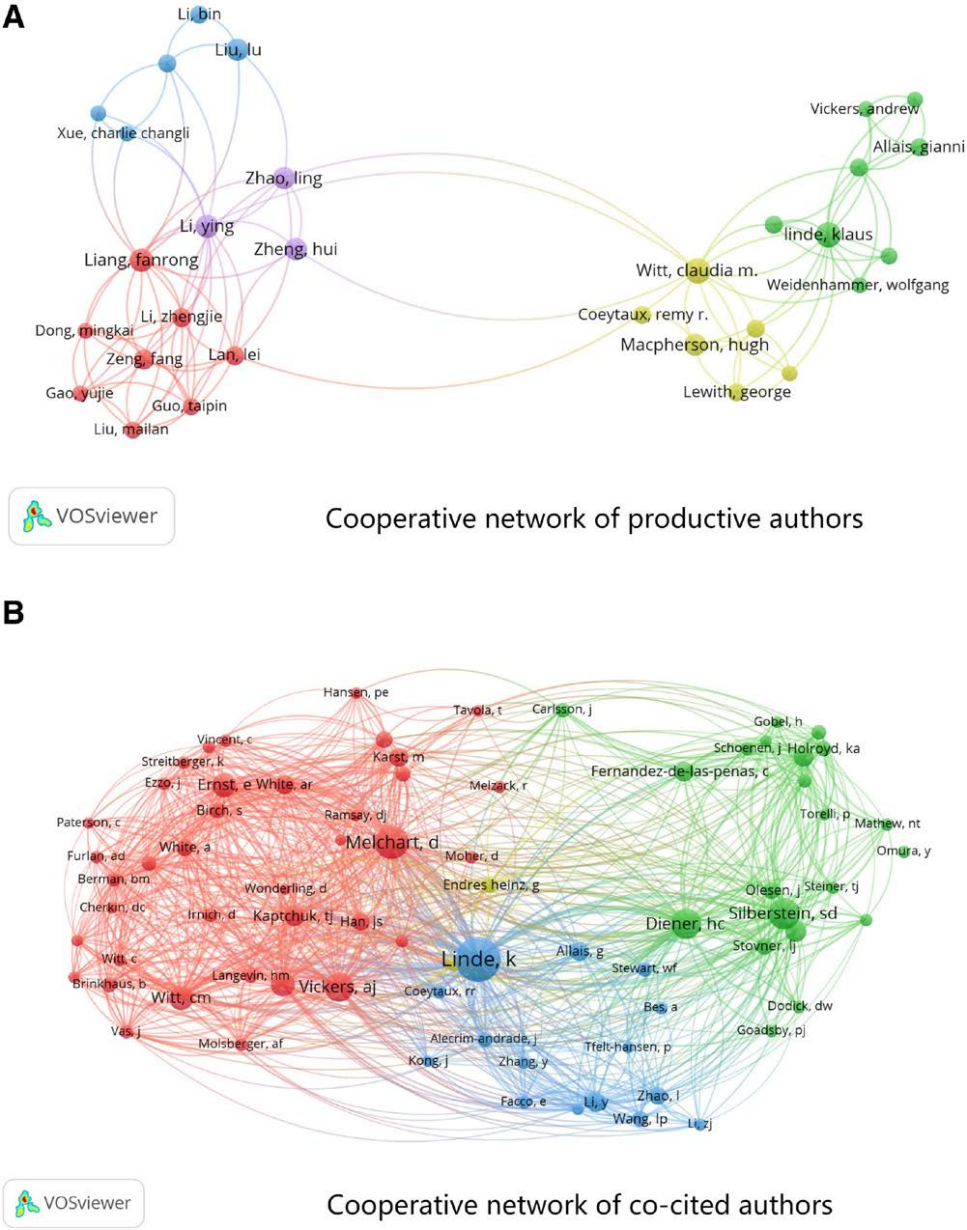
Cooperative network of productive (A) and co-cited (B) authors.

VOSviewer established a cooperative network of authors with 30 nodes and 132 links to acupuncture for headaches, as shown in Figure 4A. Each node represents an author and the size of a node is proportional to the number of publications, with a larger node indicating more published articles. Links between nodes indicate cooperation; the wider the link, the closer is the collaboration.

The top 10 productive authors in the field of acupuncture for headaches are summarized in Table 2. Linde Klaus was the most productive author in this field (33 articles), followed by Witt Claudia M (26 articles), Liang Fanrong (21 articles), Melchart D (20 articles), Li Ying (15 articles), MacPherson Hugh (15 articles), Zhao Ling (14 articles), Wang Linpeng (14 articles), Vickers Andrew J (14 articles) and Liu Lu (14 articles). Witt Claudia M (0.22) was the top centrality author, followed by Zhao Ling (0.10) and Linde K (0.09).

**Table 2:** Top ten productive and co-cited authors.

Productive author ranking	Author	Frequency (articles)	Centrality	Co-cited author ranking	Author	Frequency (citations)
1	Linde Klaus	33	0.09	1	Linde K	512
2	Witt Claudia M	26	0.22	2	Melchart D	293
3	Liang Fanrong	21	0.08	3	Silberstein SD	258
4	Melchart D	20	0.02	4	Diener HC	252
5	Li Ying	15	0.04	5	Vickers AJ	231
6	MacPherson Hugh	15	0.01	6	Witt CM	164
7	Zhao Ling	14	0.10	7	Bes A	160
8	Wang Linpeng	14	0.06	8	Macpherson H	155
9	Vickers Andrew J	14	0.05	9	Lipton RB	129
10	Liu Lu	14	0.07	10	Kaptchuk TJ	111

The network of author co-citation analysis was built using VOSviewer (Fig. 4B). Among the 13,762 coauthors, 50 (0.36%) were cited more than 50 times, 16 (0.12%) were cited more than 100 times, and 8 (0.06%) were cited at least 150 times. The top 10 co-cited authors with at least 100 citations are listed in Table 2. Linde K was the most cited author (512 citations), followed by Melchart D (293 citations), Silberstein SD (258 citations), Diener HC (252 citations), Vickers AJ (231 citations), Witt CM (164 citations), Bes A (160 citations), Macpherson H (155 citations), Lipton RB (129 citations) and Kaptchuk TJ (111 citations).

### 3.6. Analysis of journals

**Figure 5.**
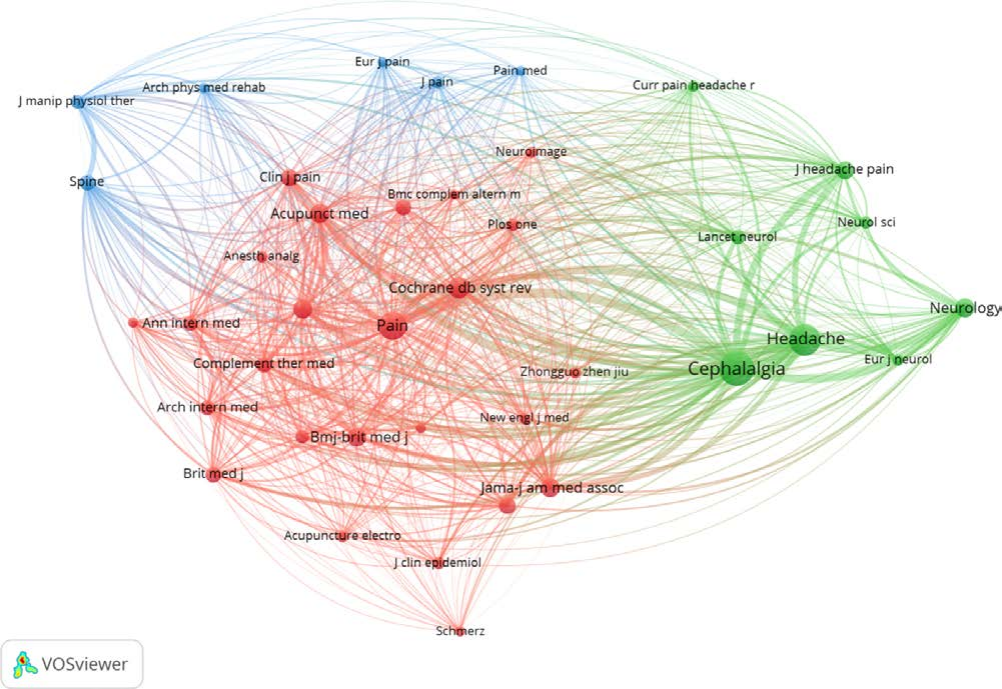
Cooperative network of co-cited journals.

VOSviewer established a cooperative network of co-cited journals with 68 nodes and 2243 links to acupuncture for headaches (Fig. 5). Each node represents a co-cited journal, and its size indicates the co-citation frequency. Links between the nodes suggest co-citation of co-cited journals, with a wider link signifying a higher frequency of co-cited journals.

The top 10 journals in the field of acupuncture for headaches are listed in Table 3. The most productive journals were Cephalalgia (34 articles), followed by Headache (32 articles), Deutsche Zeitschrift fur Akupunktur (30 articles), Evidence-based Complementary and Alternative Medicine (23 articles), Journal of Alternative and Complementary Medicine (23 articles), Acupuncture in Medicine (22 articles), Medicine (21 articles), Medical Acupuncture (19 articles), American Journal of Acupuncture (14 articles), and Trials (13 articles).

**Table 3:** Top ten productive and co-cited journals.

Ranking	Journal	Frequency (articles)	IF/Q (2022)	Ranking	Cited Journal	Frequency (citations)	Centrality	IF/Q (2022)
1	*Cephalalgia*	34	6.075/Q1	1	*Cephalalgia*	474	0.17	6.075/Q1
2	*Headache*	32	5.311/Q1	2	*Pain*	436	0.06	7.926/Q1
3	*Deutsche Zeitschrift fur Akupunktur*	30	0.000/N/A	3	*Headache*	428	0.08	5.311/Q1
4	*Evid Based Complement Alternat Med*	23	2.650/Q3	4	*JAMA*	329	0.09	157.335/Q1
5	*J Altern Complement Med*	23	2.381/Q3	5	*Cochrane Database of Systematic Reviews*	291	0.05	12.008/Q1
6	*Acupunct Med*	22	1.976/Q3	6	*BMJ*	279	0.05	93.333/Q1
7	*Medicine*	21	1.817/Q3	7	*J Altern Complement Med*	252	0.09	2.381/Q3
8	*Medical Acupuncture*	19	0.000/N/A	8	*Lancet*	249	0.48	202.731/Q1
9	*American Journal of Acupuncture*	14	5.620/Q1	9	*Neurology*	235	0.20	11.800/Q1
10	*Trials*	13	2.728/Q4	10	*Acupunct Med*	223	0.05	1.976/Q3

Evid Based Complement Alternat Med, Evidence-based Complementary and Alternative Medicine.

Acupunct Med, Acupuncture in Medicine; J Altern Complement Med, Journal of Alternative and Complementary Medicine.

BMJ = British Medical Journal, IF = impact factor, JAMA = Journal of the American Medical Association, Q = quartile, Q1 = first quartile, Q2 = second quartile, Q3 = third quartile, Q4 = fourth quartile.

The top 10 co-cited journals are listed in Table 3. Cephalalgia ranked first with 474 citations, followed by (Journal of the American Medical Association; 329 citations), and Cochrane Database of Systematic Reviews (291 citations). The highest centrality journal was Lancet (0.48), followed by Neurology (0.20), and Cephalalgia (0.17). The journal with the highest impact factor (202.731) was Lancet. Cephalalgia had the most outputs and citations, indicating that it played an important role in the field of acupuncture treatment for headaches.

### 3.7. Research hotspots and trends analysis

The research hotspots and trends in the field of acupuncture treatment of headaches were mainly analyzed using keyword co-occurrence, clustering, reference co-citation, and keyword bursts.^[15,16]^

### 3.8. Keyword analysis

**Figure 6.**
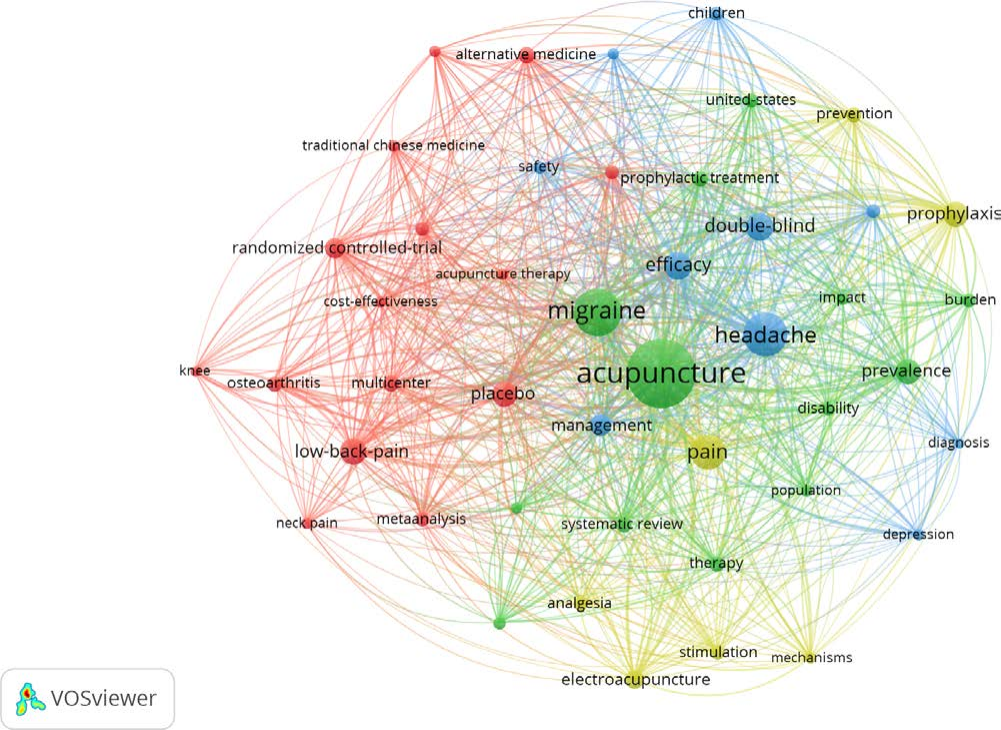
Keyword co-occurrence network.

VOSviewer built a keyword co-occurrence network with 67 nodes and 1525 links on acupuncture for headaches, as shown in Figure 6. Each node represents a keyword, and the links between nodes indicate keyword co-occurrence. The top 10 keywords in terms of frequency and centrality are listed in Table 4. Acupuncture had the highest frequency (452 times), followed by migraine (236 times), headache (218 times), pain (144 times), tension-type headache (126 times), efficacy (97 times), low back pain (92 times), double-blind (91 times), randomized controlled trial (87 times), and placebo (81 times). In terms of centrality, the highest keyword was trial (0.61), followed by pain (0.60), low back pain (0.53), electroacupuncture (0.50), and analgesia (0.43), all of which were over 0.40.

**Table 4:** Top ten keywords with centrality and frequency.

Ranking	Frequency	Keyword	Centrality	Ranking	Centrality	Keyword	Frequency
1	452	Acupuncture	0.19	1	0.61	Trial	35
2	236	Migraine	0.24	2	0.60	Pain	144
3	218	Headache	0.06	3	0.53	Low back pain	92
4	144	Pain	0.6	4	0.50	Electroacupuncture	48
5	126	Tension-type headache	0.40	5	0.43	Analgesia	19
6	97	Efficacy	0.19	6	0.40	Tension-type headache	126
7	92	Low back pain	0.53	7	0.40	Quality of life	38
8	91	Double-blind	0.33	8	0.33	Double-blind	91
9	87	Randomized controlled trial	0.07	9	0.32	Amitriptyline	5
10	81	Placebo	0.02	10	0.24	Migraine	236

The top 7 clusters (Q = 0.7319 and S = 0.7010) are summarized in Table 5. They were #0 episodic and chronic tension-type headache, #1 covid-19, #2 primary care, #3 population, #4 trigger points, #5 vertigo, #6 cervicogenic headache, and #7 placebo needle, respectively. The number order of clusters mainly depends on the size of the clusters; the smaller the label, the more keywords the cluster contains. Cluster #0 contained 17 keywords, and the first 5 feature words extracted were episodic and chronic tension-type headache, definition, pathophysiology, acute treatment, and prophylaxis. Cluster #1 included 17 members, and the first 5 features were covid-19, low back pain, neck pain, best evidence synthesis, and systematic review. Cluster #2 consisted of 15 members, and the first 5 words were primary care, acupuncture therapy/adverse effects, acupuncture/history/standards, back pain, and complementary and alternative therapy (cam). Cluster #3 contained 14 members, and the first 5 words were population, serum, ionized magnesium level, gold meridian, and controlled trial. Cluster #4 comprised of 14 members, and the first 5 were trigger points, clinical efficacy, migraine prophylaxis, Zhengtian Pill, and myofascial pain syndrome. Cluster #5 involved 13 members, and the first 5 feature words were vertigo, hemiplegia, 4-gate points, Yuan point, and stroke. Cluster #6 embraced 12 members, and the first 5 of which were cervicogenic headache, neck, tenderness, dysfunction, and chiropractic. Cluster #7 had 11 members, and the first 5 characters were placebo needle, tension headache, acupuncture research, osteoarthriti, and *deqi*.

**Table 5:** Keywords cluster.

ID Ranking (#)	Size	Silhouette	Top-term (LLR)
0	17	0.924	Episodic and chronic tension-type headache (62.98, 1.0E-4); definition (62.98, 1.0E-4); pathophysiology (59.55, 1.0E-4); acute treatment (51.01, 1.0E-4); prophylaxis (44.5, 1.0E-4);
1	17	0.882	COVID-19 (12.26, 0.001); low back pain (9.24, 0.005); neck pain (9.21, 0.005); best evidence synthesis (8.17, 0.005); systematic review (8.06, 0.005);
2	15	0.898	Primary care (13.73, 0.001); acupuncture therapy/adverse effects (9.59, 0.005); acupuncture/history/standards (9.59, 0.005) back pain (9.24, 0.005); complementary and alternative therapy (cam) (9.24, 0.005)
3	14	0.872	Population (8.11, 0.005); serum (8.11, 0.005); ionized magnesium level (8.11, 0.005); gold meridian (8.11, 0.005); controlled trial (8.11, 0.005)
4	14	0.945	Trigger points (11.68, 0.001); clinical efficacy (10.68, 0.005); migraine prophylaxis (10.68, 0.005); zhengtian pill (10.68, 0.005); myofascial pain syndrome (10.68, 0.005)
5	13	0.931	Vertigo (15.91, 1.0E-4); hemiplegia (15.91, 1.0E-4); 4-gate points (9.53, 0.005); yuan point (9.53, 0.005); stroke (9.53, 0.005)
6	12	0.835	Cervicogenic headache (19.45, 1.0E-4); neck (13.83, 0.001); tenderness (13.83, 0.001); dysfunction (13.83, 0.001); chiropractic (12.13, 0.001)
7	11	0.899	Placebo needle (17.67, 1.0E-4); tension headache (11.76, 0.001); acupuncture research (11.76, 0.001); osteoarthriti (9.68, 0.005); deqi (8.05, 0.005)

**Figure 7.**
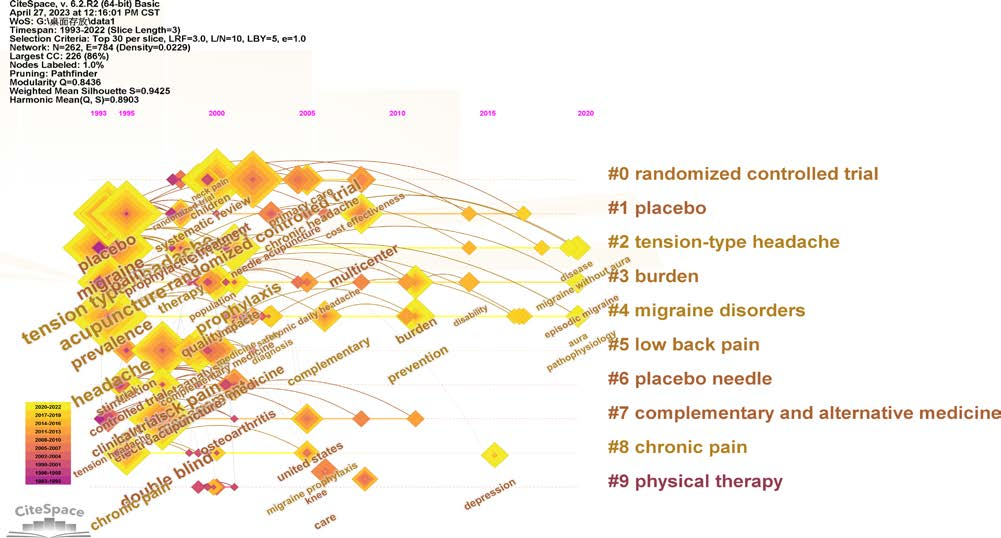
Timeline viewer of keywords cluster.

The timeline viewer of keyword clustering was performed using the CiteSpace software (Fig. 7) with a Q value of 0.8903 and an S value of 0.9425. It visually displays phased hotspots of acupuncture for headaches from a time perspective. From 1974 to 1998, the research focused on the types and prevention of headaches, and the main keywords involved were migraine, chronic headache, tension headache, migraine prevention, and physical therapy. From 1999 to 2008, the study focused on randomized controlled trials, placebo acupuncture, and complementary and alternative medicine, and main keywords included randomized controlled trial, meta-analysis, alternative medicine, complementary medicine, and placebo acupuncture. From 2009 to 2015, the study focused on placebo use, and the main keywords included placebo, tricyclic antidepressants, and serotonin reuptake inhibitors. From 2016 to 2022, the study mainly investigated the methods and effects of acupuncture and moxibustion in treating different types of headaches. The main keywords used electroacupuncture, acupuncture, trigger points, quality of life, episodic migraine, aura migraine, and cluster headache. In conclusion, the study of acupuncture treatment for headache were different in different time dimensions. Among these, the time spans of tension headache, migraine, and headache burden experiences were longer. Subsequently, the study of headache changed from initial prevention to treatment. In general, the hotspots of research are mainly focused on migraine, tension headache, electroacupuncture therapy, acupuncture effect and therapeutic efficacy, acupuncture trigger point, and placebo.

### 3.9. Co-cited reference analysis

The top 5 frequently co-cited references are presented in Table 6. “Acupuncture for Patients With Migraine: A Randomized Controlled Trial” is the most cited article in the field of acupuncture treatment for headache, with 89 citations.^[17]^ It was published on the Journal of American Medical Association in 2005, focusing on the efficacy of acupuncture in migraine patients.^[17]^ The results showed that acupuncture was no more effective than sham acupuncture in reducing migraine in a randomized controlled trial, but both interventions were more effective than a waiting-list control.^[17]^ These results provide evidence that acupuncture can be used to prevent migraines.

**Table 6:** Top five frequently co-cited references.

Ranking	Title	Author	Journal	Frequency	Year
1	Acupuncture for patients with migraine a randomized controlled trial	Linde K	*JAMA-Journal of the American Medical Association*	89	2005
2	Acupuncture for migraine prophylaxis	Linde K	*Cochrane Database of Systematic Reviews*	69	2009
3	Efficacy of acupuncture for the prophylaxis of migraine: A multicentre randomized controlled clinical trial	Diener HC	*Lancet Neurology*	65	2006
4	Acupuncture in patients with tension-type headache: Randomized controlled trial	Melchart D	*BMJ-British Medical Journal*	63	2005
5	Acupuncture for chronic headache in primary care: Large, pragmatic, randomized trial	Vickers AJ	*BMJ-British Medical Journal*	41	2004

The top 5 co-cited references with high centrality are presented in Table 7. “Cost-effectiveness of acupuncture treatment in patients with headache” had the highest centrality of 0.50.^[18]^ It was published by Witt Claudia M in Cephalalgia in 2008. This study evaluated the cost-effectiveness of acupuncture for the treatment of headache.^[18]^ The results showed that acupuncture is a cost-effective treatment for patients with primary headache, according to international cost-effectiveness threshold values.^[18]^

**Table 7:** Top five co-cited references with high centrality.

Ranking	Title	Author	Journal	Centrality	Year
1	Cost-effectiveness of acupuncture treatment in patients with headache	Witt CM	*Cephalalgia*	0.50	2008
2	Efficacy of acupuncture for migraine prophylaxis: A single-blinded, double-dummy, randomized controlled trial	Wang LP	*Pain*	0.26	2011
3	Effects of long-term acupuncture treatment on resting-state brain activity in migraine patients: A randomized controlled trial on active acupoints and inactive acupoints	Zhao L	*PloS One*	0.22	2014
4	Acupuncture in migraine prophylaxis: A randomized sham-controlled trial	Alecrim-Andrade J	*Cephalalgia*	0.21	2006
5	Cerebrovascular response in migraineurs during prophylactic treatment with acupuncture: A randomized controlled trial	Wallasch TM	*Journal of Alternative and Complementary Medicine*	0.16	2012

### 3.10. Research trend analysis

**Figure 8.**
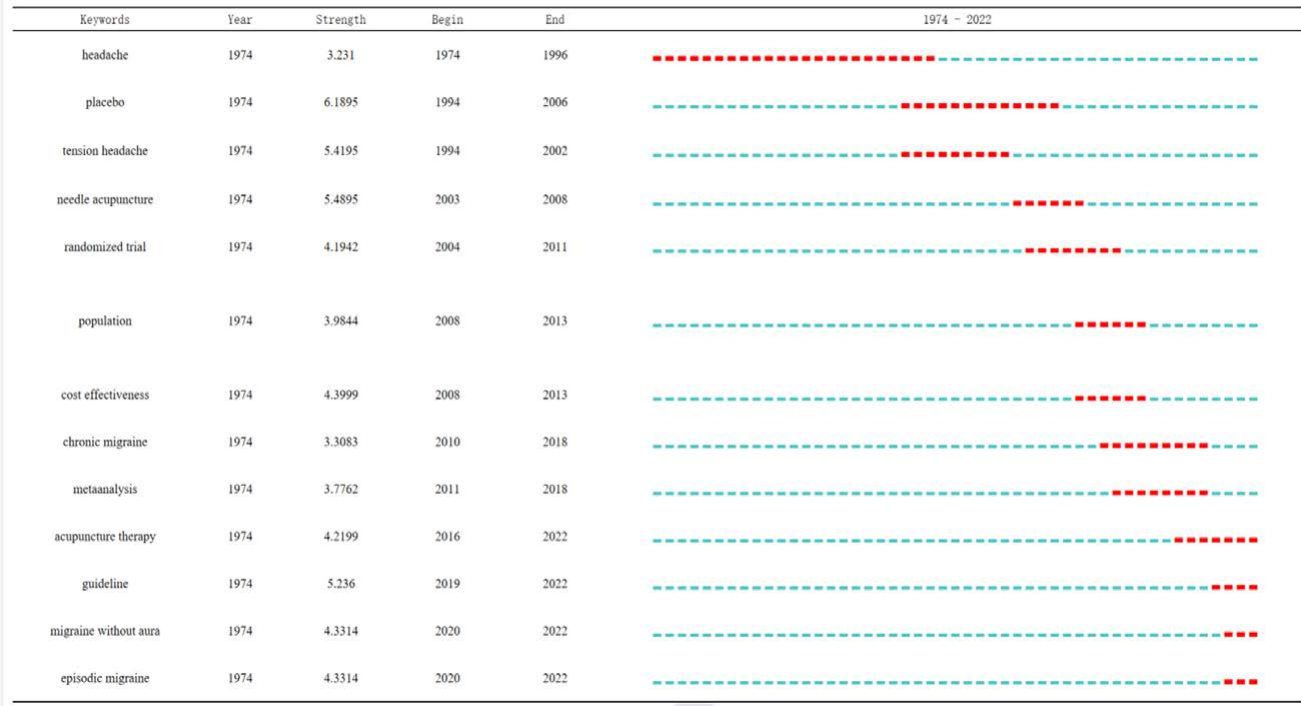
Top 13 keywords with the strongest citation bursts.

Keywords with the strongest citation bursts by CiteSpace are listed in Figure 8. The primary setting in the process is a Minimum Duration of 2 years. In the table, Begin and End indicate the start and end times of the bursts, respectively, and Red indicates the time span of the bursts.

The main set in the process is a minimum duration of 1 year and a γ of 3.33. In the graph, Begin and End indicate the start and End times of the burst, respectively, and the red part indicates the time span of burst. As can be seen from the table, headache has been bursted since 1974 and lasted until 1996 with a burst intensity of 3.231. It had the longest burst duration, indicating that headache is of great importance for research in this field. A placebo, which is commonly used as a control in trials, is an important reference for trial results. The second keyword, acupuncture needle, had a burst intensity of 5.4895. “Acupuncture therapy,” “guideline,” “migraine without aura” and “episodic migraine” were bursted from the years of 2016 to 2022, 2019 to 2022, 2020 to 2022, and 2020 to 2022, respectively, and they have continued to the present. These are current research hotspots and trends for future research.

## 4. Discussion

The data for this study were obtained from the WOSCC database from 1974 to 2022 using CiteSpace and VOSviewer software. The countries, institutions, authors, journals, keywords, and references in this field are displayed visually; the relevant references are comprehensively analyzed; and the research status, hotspots, and trends in this field are investigated.

### 4.1. Research status of acupuncture for headache

In terms of publications, the number of published studies in this field shows an increasing trend as a whole, and the change in publications is presented as y = 13.914 ln(x) to 23.887, with an annual number of publications of 17.5. Thus, the overall degree of attention increased. The United States had the highest output of literature, accounting for 27.94% of the total publications. This was followed by China, Germany, the United Kingdom, Italy, and Switzerland, which had relatively high-output publications. China, Germany, the United States, and the United Kingdom have a high centrality and great influence. The close connection of cooperative networks among countries has laid a good foundation for the development of this field.

Among the institutions, Chengdu University of Traditional Chinese Medicine had the most output publications, followed by the Technical University of Munich, Beijing University of Chinese Medicine, Memory Sloan Kettering Cancer Center, Capital Medical University, and University of Maryland. Chengdu University of Traditional Chinese Medicine, Technical University of Munich, and Beijing University of Chinese Medicine had higher centrality. These results are of great significance for the development of this field. Cooperation and close relationships of collaboration among high-yield institutions is a most promising development in this field.

Authors in this field have formed good cooperative relationships, and their collaboration is mainly among high-yield authors. Linde Klaus was the most prolific author, accounting for 3.92% of the total publications. Research mainly involves the curative effect of acupuncture on chronic pain, such as migraine and tension headache,^[19,20]^ prevention of paroxysmal migraine by acupuncture,^[21]^ and treatment of tension-type headache by acupuncture.^[22]^

Witt Claudia M had the highest centrality. Her research mainly focused on the effect of patient characteristics on acupuncture treatment outcomes^[23]^ and physician characteristics and variations in treatment outcomes of chronic pian by acupuncture.^[24]^ Authors such as Liang Fanrong, Melchart D, and Zhao Ling have significantly contributed to the development of this field.

In terms of journals, Cephalalgia was the most output frequently cited journal, accounting for 4.04% of the total papers, and the impact factor (IF-2022) was 6.075 within the first quartile. The overall average IF of the first ten productive journals was 2.856, and the ratio of first quartile was 30%. The overall average IF of the first ten journals cited was 50.088, and the ratio of Q 1 was 80%. Lancet was the most co-cited journal in the field, with a highest IF of 202.731. The journal with the highest centrality was Lancet (0.48), followed by Neurology (0.20) and Cephalalgia (0.17).

### 4.2. Research hotspots and trends on acupuncture for headaches

According to keyword co-occurrence, acupuncture, migraine, headache, pain, tension-type headache, efficacy, low back pain, double-blind, randomized controlled trial, and placebo were high-frequency keywords in the research field of acupuncture treatment for headache. With the regard to centrality, the keywords trial, pain, lower back pain, electroacupuncture, analgesia, tension-type headache, quality of life, double-blind, amitriptyline, and migraine were the top 10, and the most influential, and were all over 0.10. In terms of keyword clustering, they are scientific, reasonable, and reliable, whether as a whole or individually. They were divided into 8 types: #0 episodic and chronic tension-type headache, #1 covid-19, #2 primary care, #3 population, #4 trigger points, #5 vertigo, #6 cervicogenic headache, and #7 placebo needle. It can be concluded that headache, migraine, tension headache, electroacupuncture, acupuncture effect and curative effect, acupuncture trigger point, and placebo acupuncture are major research hotspots in this field. Double-blind randomized controlled trials are the most common research method in this field.

In light of co-cited references, the top 5 co-cited and high centrality references were published in the field of international journals with high IF. They have an important influence and academic reference value. Among these, the most frequently co-cited references mainly involved the effect of acupuncture for migraine prophylaxis,^[17,25]^ evidence of acupuncture for the prophylaxis of migraine,^[26]^ acupuncture in patients with tension-type headache,^[27]^ and acupuncture for chronic headache in primary care.^[28]^

The high centrality articles mainly involved the cost and cost-effectiveness of acupuncture in the treatment of headache patients.^[18]^ Acupuncture was more effective than flunarizine in reducing the number of days of migraine attacks,^[18]^ there was no significant difference between acupuncture and flunarizine in reducing pain intensity and improving quality of life,^[29]^ effect of long-term acupuncture on resting brain activity of migraine patients,^[30]^ no difference between semi-standardized acupuncture and sham acupuncture in the prevention of migraine attacks,^[31]^ and acupuncture prevention and treatment of migraine may have a positive effect on the disturbance of cerebral vascular response to spontaneous stimulation, but not on cerebral vascular access at rest.^[32]^

According to the keyword bursts, headache had the longest time span burst, and placebo had the strongest burst at 6.1895. Acupuncture therapy, guideline, migraine without aura (MWoA), and episodic migraine were bursted from 2016 to 2022, 2019 to 2022, 2020 to 2022, and 2020 to 2022, respectively, and they lasted to the present. These are the current research hotspots and trend for future research. Zhao Zheng reported that acupuncture treatment for 4 weeks could significantly reduce the days and duration of headache attack, and had a preventive effect on patients with chronic migraine. Among them, the therapeutic effect of acupuncture therapy in the prevention and treatment of chronic migraine is significant.^[33]^ Zhang Kai reported a multicenter randomized controlled clinical trial of 3-step acupuncture and cupping therapy for cervicogenic headaches.^[34]^ The results showed that 3-step acupuncture and cupping were more effective, less recurrent, and safer than sham acupuncture in the treatment of cervicogenic headache.^[34]^ Xing Xiao reported that in treatment of primary headache immediate analgesic effect, *GaoYanzhao DuMaiTongLuo* acupuncture method was superior to the ordinary acupuncture method.^[35]^ Supasiri Thanan compared the effects of different sessions of acupuncture on migraine. The results showed that 5 and 10 sessions of acupuncture had significant benefits in preventing migraine attacks, reducing the severity of headache, and improving quality of life.^[36]^ Dunning James pointed out that electrical dry needling had a better effect on patients with cervicogenic headache.^[37]^ Liu Lu reported that acupuncture has become a promising treatment for MWoA. Liu has used resting-state-functional magnetic resonance imaging (MRI) to study the brain mechanisms of acupuncture.^[38]^ The results suggest that acupuncture may modulate the seed-voxel resting-state-functional connectivity of 2 pain-regulating regions in the right amygdala and right middle cingulate cortex simultaneously, and the middle temporal gyrus and the right superior temporal gyrus may be the key nodes related to the multi-sensory processing of pain regulation in acupuncture treatment of migraine.^[38]^ Liu Shanshan found that acupuncture relieved migraine symptoms, while also improving cerebellar dysfuntion, and that acupuncture activated areas of the brain associated with pain and mood regulation.^[39]^ Interestingly, the cumulative therapeutic effect of acupuncture was found to be more robust and significant than its direct effect.^[39]^ Hong Jiahui study used multimodal MRI scans to study the mechanisms of acupuncture at baseline, at the end of treatment, and after follow-up.^[40]^ Using machine learning technology, multimodal MRI data can be used to predict the therapeutic effect of acupuncture, but there is still a gap in the study of the neural mechanism of acupuncture in treating MWoA.^[40]^ Naguit Noreen study found that acupuncture could be used as an alternative or adjunctive therapy for the treatment of episodic headache.^[41]^ Beier Dagmar study provided an updated guide to some of the most widely used non-pharmacological treatments for migraine.^[42]^ Guidelines suggest that physical activity may have a positive effect on quality of life, with acupuncture having a positive impact on headache frequency, intensity, quality of life, and aggressive drug use.^[42]^ Patient education may improve the quality of life and increase the number of informed patients.^[42]^

Overall, future research in the field of acupuncture treatment of headache will mainly focus on the study of acupuncture therapy and its curative effect, MWoA, paroxysmal migraine, the mechanism of acupuncture treatment, and the development and innovation of related guidelines for acupuncture treatment of headache. In addition, the use of multimodal MRI will be of great help in the study of the neural mechanism of acupuncture in the treatment of headaches.

## 5. Limitations

This study has several limitations. First, all included studies were obtained from the WOSCC database, which cannot ensure a comprehensive coverage of the literature. Second, the records were obtained from 1974 to 2022. However, with the continuous update of WOSCC, the search results may not be similar to the actual number of eligible records. Third, the inconsistent quality of the included records may have decreased the credibility of the map analysis. Fourth, this study only analyzed records published in English; thus, publications in other languages were not included. Fifth, some potentially eligible publications may have been lost because of their manual official reports. Finally, CiteSpace and VOSviewer softwares have a certain threshold setting in the visualization process; to some extent, they fail to pay sufficient attention to low-frequency, low-centerality and low-cited references. In other words, they focused on revealing major parts of the field.

## 6. Conclusion

In this study, we investigated research hotspots and frontier trends in acupuncture treatment for headache from 1974 to 2022. This study has shown great potential in the field of acupuncture treatment for headache. Research hotspots have mainly focused on headache, migraine, tension headache, electroacupuncture, acupuncture effect and curative effect, acupuncture trigger points, and placebo. Future research trends are mainly embodied in the research on acupuncture therapy and its curative effect, MWoA, paroxysmal migraine, the mechanism of acupuncture therapy, and the development and innovation of related guidelines for acupuncture treatment of headache. In addition, the use of multimodal MRI will be of great help in the study of the neural mechanism of acupuncture in the treatment of headache.

## Author contributions

**Conceptualization:** Jin-Huan Yue, Ang Li, Xiao-Ling Li, Qinhong Zhang.

**Data curation:** Jin-Huan Yue, Ang Li, Xu-Chen Sun, Xu Yang, Xiao Liu, Wei-Wei Zhao, Yang Wang, Qin-Hong Zhang.

**Formal analysis:** Ang Li, Xu-Chen Sun, Xu Yang.

**Funding acquisition:** Xiao-Ling Li, Dan-Na Cao, Sheng-Wang Han.

**Investigation:** Xiao-Ling Li, Qin-Hong Zhang.

**Methodology:** Ang Li, Xu Yang, Qin-Hong Zhang.

**Project administration:** Xiao-Ling Li, Guanhu Yang, Qin-Hong Zhan.

**Resources:** Xuan Cui, Xu-Chen Sun, Xu Yang, Dan-Na Cao, Guan-Hu Yang, Yang Wang, Sheng-Wang Han.

**Software:** Xu-Chen Sun, Xu Yang, Yang Wang.

**Supervision:** Xiao-Ling Li, Qin-Hong Zhang.

**Validation:** Jin-Huan Yue, Ang Li, Xuan Cui, Xu-Chen Sun, Xiao-Ling Li, Xu Yang, Xiao Liu, Dan-Na Cao, Wei-Wei Zhao, Guan-Hu Yang, Brenda Golianu, Yang Wang, Sheng-Wang Han, Qin-Hong Zhang.

**Visualization:** Jin-Huan Yue, Ang Li, Xuan Cui, Xu-Chen Sun, Xiao-Ling Li, Xu Yang, Xiao Liu, Dan-Na Cao, Wei-Wei Zhao, Guan-Hu Yang, Brenda Golianu, Yang Wang, Sheng-Wang Han, Qin-Hong Zhang.

**Writing – original draft:** Jin-Huan Yue, Ang Li, Xuan Cui, Xu-Chen Sun, Xiao-Ling Li, Xiao Liu, Dan-Na Cao, Wei-Wei Zhao, Guan-Hu Yang, Brenda Golianu, Yang Wang, Sheng-Wang Han, Qin-Hong Zhang.

**Writing – review & editing:** Jin-Huan Yue, Ang Li, Xuan Cui, Xu-Chen Sun, Xiao-Ling Li, Xu Yang, Xiao Liu, Dan-Na Cao, Wei-Wei Zhao, Guan-Hu Yang, Brenda Golianu, Yang Wang, Sheng-wang Han, Qin-Hong Zhang.
